# An Economic Analysis for the Use of Artificial Intelligence in Screening for Diabetic Retinopathy in Trinidad and Tobago

**DOI:** 10.7759/cureus.55745

**Published:** 2024-03-07

**Authors:** Ryan R Ramoutar

**Affiliations:** 1 Ophthalmology, University Hospitals of Leicester NHS Trust, Leicester, GBR

**Keywords:** cost-benefit, screening, diabetic retinopathy, diabetes, health management, epidemiology, public health, artificial intelligence in ophthalmology

## Abstract

This is a systematic review of 25 publications on the topics of the prevalence and cost of diabetic retinopathy (DR) in Trinidad and Tobago, the cost of traditional methods of screening for DR, and the use and cost of artificial intelligence (AI) in screening for DR. Analysis of these publications was used to identify and make estimates for how resources allocated to ophthalmology in public health systems in Trinidad and Tobago can be more efficiently utilized by employing AI in diagnosing treatable DR. DR screening was found to be an effective method of detecting the disease. Screening was found to be a universally cost-effective method of disease prevention and for altering the natural history of the disease in the spectrum of low-middle to high-income economies, such as Rwanda, Thailand, China, South Korea, and Singapore. AI and deep learning systems were found to be clinically superior to, or as effective as, human graders in areas where they were deployed, indicating that the systems are clinically safe. They have been shown to improve access to diabetic retinal screening, improve compliance with screening appointments, and prove to be cost-effective, especially in rural areas. Trinidad and Tobago, which is estimated to be disproportionately more affected by the burden of DR when projected out to the mid-21st century, stands to save as much as US$60 million annually from the implementation of an AI-based system to screen for DR versus conventional manual grading.

## Introduction and background

Gregory et. al found that the global prevalence of type 2 diabetes in adults in 2021 was estimated to be 536 million people (10.5%), with a projection to grow to 783 million in 2045 (12.2%), while Sun et. al found that the global prevalence of type 1 diabetes in the same year was estimated at 8.4 million with a projection to grow to 15.5 million by 2040 [1,2].

Diabetic retinopathy (DR) is the major ocular complication of diabetes mellitus and is a leading cause of blindness and visual impairment in the working-age population globally, with more than 100 million people (30-40% of diabetics) living with some form of DR [3-5].

The global prevalence and disease burden of DR is expected to increase significantly during the middle of the 21st century, from about 100 million individuals in 2020 to 130 million in 2030 and 161 million in 2045 [6]. The International Diabetes Federation in 2022 estimated the prevalence of diabetes in the adult population in Trinidad and Tobago was 14.8%, more than double the estimated global burden. DR was determined by Braitwaite et al. to account for 19% of blindness in adults over 40 years old in Trinidad and Tobago [7]. An estimated 9.5% of the national budget of Trinidad and Tobago was spent on eye care in 2014 [8].

Wong et al., Vijan et al., and James et al. produced studies corroborating that screening for DR is considered both a primary and secondary prevention method and has been proven to be a cost-effective means of reducing the burden of disease to public health systems [9-12]. Screening for disease is an important health management tool and has been formalized by the World Health Organization. DR screening began as a pilot project at the South-West Regional Health Authority in Trinidad and Tobago in 2013 and, after a successful introduction, had screened 10,000 people by 2018.

The emergence of artificial intelligence (AI) and deep learning algorithms for screening DR has led to cost reductions of up to 20% in screening and has improved resource utilization in managing the health burden of diabetes [13-15].

The aim of this paper is to explore the current use of AI for DR screening around the world and to identify whether the introduction of AI for DR screening could improve resource utilization in the public health sector of Trinidad and Tobago. 

The main objectives of this review are as follows:

(i) To identify how AI is currently used in the diagnosis and management of DR in countries where it has been adopted

(ii) To analyze how AI can lead to more efficient utilization of resources in screening for DR

(iii) To recommend whether the use of AI for the diagnosis and management of DR should be introduced in the health system of Trinidad and Tobago

Method of analysis

This study is a systematic review undertaken to identify how resources allocated to ophthalmology in the public health system of Trinidad and Tobago can be more efficiently utilized by employing AI in diagnosing treatable DR, using established models in other countries.

A literature search was conducted on articles indexed on PubMed, Google Scholar, and Cochrane Review search engines, which were searched for between May 2023 and July 2023. Journal articles were limited to publication within the last 10 years unless such paper was a ‘landmark’ paper on which future, significant research is based. Keywords used in the search included ‘ophthalmology’, ‘diabetic retinopathy’, ‘screening’, ‘artificial intelligence’, ‘deep learning’, ‘Trinidad’ and ‘Trinidad and Tobago’. These keywords were used as single search terms or in combination.

Gaps in the review include a paucity of studies conducted in Trinidad and Tobago and the wider Caribbean on the impact of DR and DR screening. A further gap has been identified on the basis of no literature available for the use of AI or deep learning systems for screening programs in Trinidad and Tobago.

Inclusion criteria

(i) Papers published within the last 10 years

(ii) Papers that specifically mention AI and its use in detecting DR

(iii) Papers that include an analysis of the impact of DR on health systems and resources

(iv) Papers that include an analysis of how using AI and deep learning in the diagnosis and management of DR can more efficiently allocate resources within health systems

(v) Papers that analyze the impact of DR on the health system in Trinidad and Tobago

(vi) Papers must be peer-reviewed

(vii) Article text must be fully accessible

(viii) Papers must be written in English

Exclusion criteria

(i) Papers published more than 10 years ago

(ii) Papers not published in English or where an English translation is not available

(iii) Papers where there is only partial or abstract-only access

(iv) Non-peer-reviewed articles

## Review

**Figure 1:**
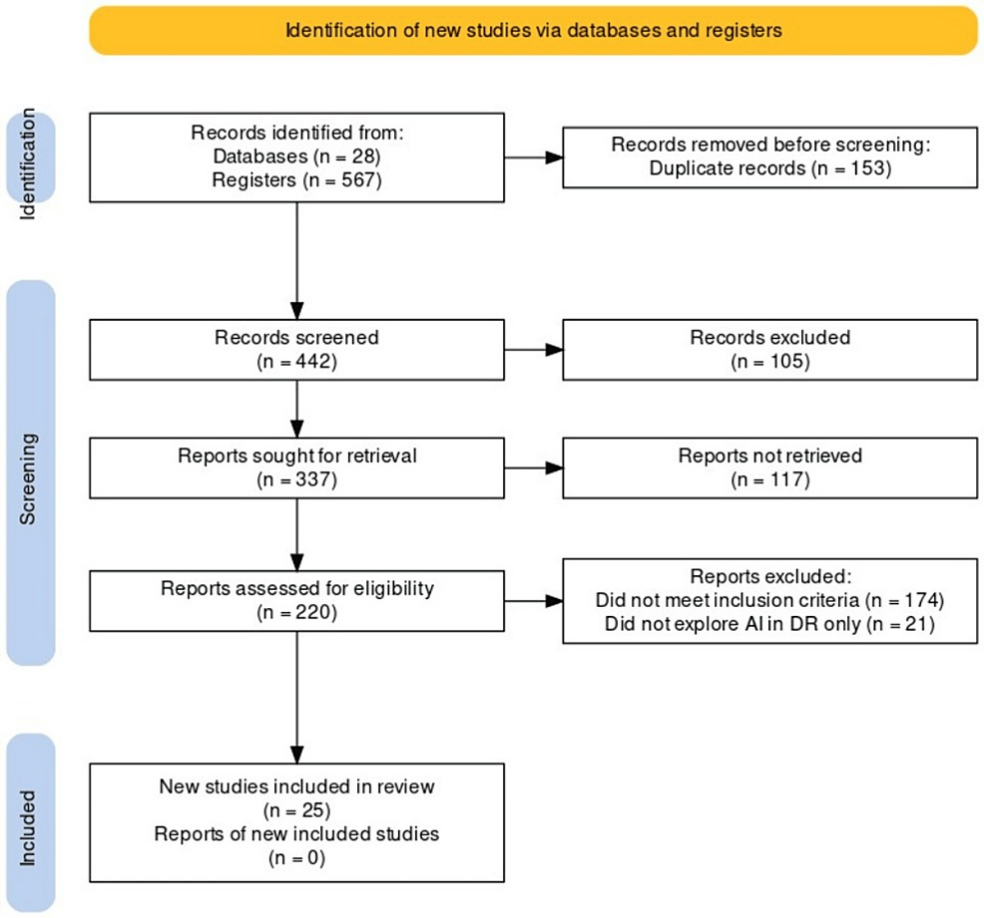
PRISMA flow diagram used for this review

Figure 1 shows the Preferred Reporting Items for Systematic Reviews and Meta-Analyses (PRISMA) flow diagram used to select the articles for this review [16].

A cross-sectional analysis of 43 studies conducted by Zhelev et al. looked at deep learning and machine learning for screening DR and showed that AI-based systems are more sensitive than human graders and could be safe to use in clinical practice [17]. Recent studies by Huang et al. and Nguyen et al. have shown that telemedicine and AI-based screening for DR are more cost-effective than human-based screening [18,19].

Cost-effectiveness of DR screening

Kim et al. used Markov models to determine the cost utility of DR screening in South Korea and concurred with other studies that showed that systematic examination for DR was the most cost-effective [11,20,21]. They estimated the cost to the Korean healthcare system at US$384,000 per 10,000 diabetics screened. Thomas et al. described resource utilization of screening and the impact of DR treatment in Wales [22]. Derived from multiple sources, the data show that the cost of hospital-based screening was approximately one-tenth of the cost of surgical intervention (vitrectomy) and less than 2% of the cost of sight loss to a person living in a care home. This is strong evidence that screening for DR is a clinical and cost-effective means of preventing disease progression. This finding has been further validated by a systematic review of other studies such as that conducted by Avidor et al. [23].

Impact of DR on the health system of Trinidad and Tobago

The National Eye Survey of Trinidad and Tobago (NESTT) estimated that approximately 9.5% of healthcare expenditure in Trinidad and Tobago was allocated to ophthalmology in 2014 and that 19% of blindness in Trinidad and Tobago was as a result of DR [7].

The Diabetic Retinal Screening Programme of Trinidad and Tobago (DRSTT) was piloted at the South-West Regional Health Authority (SWRHA) in 2013 and, since inception, has screening 10,000 people at a cost of TT$2 million (US$294,000). The cost of DR screening to the health system in Trinidad and Tobago was estimated to be US$5.7 million or 0.65% of the overall allocation to healthcare in the 2020 National Budget. This figure accounts for screening only and does not factor in other mechanisms of resource consumption such as treatment or visual loss due to DR. At the time of its rollout, only one Regional Health Authority (RHA) in Trinidad and Tobago, with a catchment of approximately 40% of the overall population, conducted DR screening in the public sector. The cost savings to the public health system in Trinidad and Tobago could be magnified since there is under-screening of the diabetic population for which more costly interventions would be required, as described by Thomas et al. [22].

The use of AI in screening for DR

Jiang et al. described medical AI as a system that is capable of 'learning' patterns and features from clinically annotated datasets, and using these insights to assist in clinical practice [24]. The nature of DR screening, with the use of color fundus photos to create datasets, lends itself as particularly amenable to deep learning and AI systems. 

Deep learning, a type of AI, has been retrospectively and prospectively validated as an effective tool for screening DR, with levels of performance on par with expert ophthalmologists [25-28]. Mathenge et al., in a robust, randomized controlled trial (RAIDERS) conducted in Rwanda, found that an important benefit of AI screening for DR was improved adherence to treatment [29].

The adoption of AI systems for DR screening in healthcare is a new and innovative form of digital technology management. It leans heavily on existing hardware infrastructure, and the cost of software has been estimated at around 7% of the overall cost of screening, according to Lin et al. [13]. Since 2018, the Food and Drug Administration (FDA) of the Unites States of America has approved a number of AI devices for screening for DR. Pei et al. found that EyeWisdom^TM^DSS was at least as effective as human ophthalmologist screeners and would have utility in low-income settings, while Zhao et al. established that the XGBoost deep-learning model of screening for DR can detect retinopathy up to three years earlier than manual screening [30,31]. Lim et al. found that the EyeArt system was far more sensitive in detecting retinopathy than human graders, validating the findings of Ipp et al. two years earlier [32,33].

Vujosevic et al. posited that DR screening assisted by AI could change the concept of screening to not just prevent sight-threatening disease but also extend to other diabetic complications, such as patients at risk of cardiovascular disease or cognitive impairment [34].

AI in DR screening - resource utilization

Markov decision-analytic models have been used by multiple researchers as the standard to estimate and compare costs and outcomes of DR screening [14,15,19]. Chawla et al. used a Markov decision-analytic model to compare the automated versus manual screening and management pathway for diabetic patients with unknown retinopathy status in the United States (a high income country) [14]. This fully automated retinal image screening (FARIS) model demonstrated cost savings of 18.8% at five years with equivalent net quality-adjusted life year (QALY) gains to manual screening. Pietris et al. undertook a systematic review to determine the economic impact of AI screening for DR in Australia and suggested that, in the Australian setting, the use of AI in combination with teleophthalmology has the potential to reduce the burden of false-positive referrals and those triaged to tertiary care, thereby incurring cost savings [35].

Srisubat et al. also utilized the Markov decision-analytic model to estimate lifetime costs and outcomes of Thailand’s (a middle-income country) national DR screening program via deep learning and trained human graders. This model showed a benefit of US$ 20 to US$ 50 per patient, depending on compliance [15].

Finally, Lin et al. set out to measure if AI-led grading for DR could lead to a reduction in cost in low- and middle-income countries (LMICs) [13]. They acknowledged that existing studies in high-income countries showed a reduction in cost by 20% from implementing AI-led screening for DR and attributed the majority of this reduction to the high labor cost of hiring human graders in high-income countries [36,37]. The research was based in Shanghai and utilized a Markov decision-analytic model. It showed that there was no cost-benefit to the implementation of AI in diabetic screening in Shanghai and attributed this to the low cost of human labour. They further demonstrated that no cost-benefit would accrue even if the AI software was free as that aspect of capital outlay only accounted for 7% of the overall cost. This finding is consistent with the findings of another systematic review, which cast doubt on the cost-effectiveness of AI screening for DR in low-income settings [38]. For the purposes of projections and estimates from this review, Trinidad and Tobago is treated as a high-income country (a designation of the World Bank since 2006), which also expends a large proportion (7.31% in 2020) of its gross domestic product (GDP) on health [39].

The model described by Huang et al. can be used as the basis for improving resource utilization by introducing AI for DR screening in Trinidad and Tobago [18]. Ipp et al., in a robust multicentre trial in the United States, showed that improved access to reliable diabetic eye exams may improve compliance with screening intervals, allowing for earlier referrals of patients deemed to have retinopathy requiring treatment [33]. These additional benefits modify the natural history of the underlying disease and may have implications for prevention of other effects of diabetes, such as renal failure, heart failure, and diabetic foot care. Modifying the course of the disease by early and accurate detection via AI or deep learning systems would have an additional cost-savings effect, thereby further leading to more efficient resource management.

Adopting the cost savings of 10% per diabetic patient from the time of diagnosis as derived by Huang et al., the extrapolated cost savings from AI in DR Screening in Trinidad and Tobago, based on the current prevalence of diabetes, can exceed US$60 million annually (20% of current population of approximately 1.5 million people and annual cost savings of US$200 per person) [18].

Table 1 summarizes the 25 articles and brief findings used in this review.

**Table 1:** Summary of the key findings from the articles used in this review

	AUTHOR (YEAR)	THEME	TOPIC	STUDY DESIGN	KEY FINDING(S)
1	Teo et al. 2021 [6]	Epidemiology	Global prevalence of diabetic retinopathy and projection of burden through 2045	Systematic analysis (n=59)	Global DR population will increase by 55.6% (57.4 million) from 2020 to 2045; Diabetic retinopathy prevalence was highest in Africa (35.90%) and North American and the Caribbean (33.30%) and was lowest in South and Central America (13.37%).
2	Braithwaite et al. 2020 [7]	Epidemiology	National Eye Survey of Trinidad and Tobago (NESTT): prevalence, causes and risk factors for presenting vision impairment in adults over 40 years	Population survey (n=2,790)	Trinidad and Tobago's burden of avoidable VI exceeds that of other high-income countries; Diabetic retinopathy was the 2^nd^ leading cause of blindness in the population, accounting for 19.1% of all blindness
3	Braithwaite et al. 2018 [8]	Epidemiology	Health system dynamics analysis of eyecare services in Trinidad and Tobago and progress towards vision 2020 goals	National survey (n=3,074)	In 2014 weaknesses in Trinidad and Tobago’s eye care system included insufficient ophthalmic equipment and human resources in the public sector, and the absence of nationwide screening programmes for sight-threatening conditions including diabetic retinopathy, congenital ocular defects, and retinopathy of prematurity.
4	Wong et al. 2020 [9]	Public health/technology	Strategies to tackle the global burden of diabetic retinopathy	Editorial	The progress made in reducing DR blindness in high-income countries may be overwhelmed by the increasing numbers of patients with diabetes and DR in low- and middle-income countries; A paradigm shift in strategic focus from tertiary towards secondary and primary prevention measures with a multi-pronged whole-of-society approach at regional and national levels is urgently needed.
5	Lin et al. 2023 [13]	Health economics	Artificial intelligence in community-based diabetic retinopathy telemedicine screening in urban China: cost-effectiveness and cost-utility analyses with real-world data	Prospective Markov model (n= 32,695)	Value of AI in telemedicine for screening DR is linked to referral compliance
6	Srisubat et al. 2023 [15]	Health economics	Cost-utility analysis of deep learning and trained human graders for diabetic retinopathy screening in a nationwide program	Prospective Markov model (n=1,000)	DR screening using Deep Learning in a Middle Income Country, using Thailand as a model, may result in societal cost-savings and similar health outcomes compared with Human Graders
7	Zhelev et al. 2023 [17]	Public health/technology	Test accuracy of artificial intelligence-based grading of fundus images in diabetic retinopathy screening: a systematic review	Systematic review (n=43)	AI-based systems are more sensitive than human graders and could be safe to use in clinical practice; Pre-implementation assessment in the target clinical pathway is essential to obtain reliable and applicable accuracy estimates
8	Huang et al. 2022 [18]	Health economics	Cost-effectiveness of artificial intelligence screening for diabetic retinopathy in rural China	Prospective Markov model (n=1,000)	AI-based screening is more cost-effective compared with conventional ophthalmologist screening
9	Nguyen et al. 2016 [19]	Health economics	Cost-effectiveness of a national telemedicine diabetic retinopathy screening program in Singapore	Prospective Markov model (n=170,000)	Telemedicine-based DR screening using technicians in the primary care setting saves costs for Singapore compared with the Family Physician model.
10	Kim et al. 2015 [20]	Health economics	Cost-utility analysis of screening strategies for diabetic retinopathy in Korea	Prospective Markov model (n= 20,000)	Systematic photography is the best strategy for DR screening in terms of cost-utility
11	Thomas et al. 2020 [22]	Health economics	Cost-effectiveness of biennial screening for diabetes-related retinopathy in people with type 1 and type 2 diabetes compared to annual screening	Retrospective observational (n= 91,393)	Base case and sensitivity analyses indicate biennial screening to be cost-effective for T2DM irrespective of HbA1c and duration of diabetes; Cost savings as much as £106,000 per QALY by extending screening biennially based on HbA1c levels
12	Avidor et al. 2020 [23]	Health economics	Cost-effectiveness of diabetic retinopathy screening programs using telemedicine: a systematic review	Systematic review (n=7)	Diabetic retinopathy telemedicine technology has the potential to provide significant cost savings, especially in low-income populations and rural patients with high transportation costs.
13	Grzybowski et al. 2020 [25]	Public health/technology	Artificial intelligence for diabetic retinopathy screening using color retinal photographs	Systematic review	AI for DR screening should follow the governance of AI in healthcare to ensure fairness, transparency, trustworthiness, and accountability for all the stakeholders including policy-makers, healthcare providers, AI developers, and patients.
14	Wang et al. 2023 [28]	Public health	Use of artificial intelligence in diabetic retinopathy screening	Systematic review (n=21)	AI has diagnostic value when screening for DR
15	Mathenge et al. 2022 [29]	Public health	Impact of artificial intelligence assessment of diabetic retinopathy on referral service uptake in a low-resource setting: the Raiders randomized trial	Randomized controlled trial (n=823)	Important benefit of AI screening in promoting adherence to prescribed treatment for diabetic eye care in sub-Saharan Africa
16	Pei et al. 2022 [30]	Technology/health economics	Efficacy of artificial intelligence-based screening for diabetic retinopathy in type 2 diabetes mellitus patients	Prospective observational (n=563)	It is valuable to carry out AI-based DR screening in poorer regions.
17	Zhao et al. 2022 [31]	Public health/technology	Using machine learning techniques to develop risk prediction models for the risk of incident diabetic retinopathy among patients with type 2 diabetes mellitus	Retrospective cohort (n=7,943)	The model achieved high performance in predicting the risk of DR among patients with type 2 diabetes mellitus at each time point.
18	Lim et al. 2023 [32]	Public health/technology	Artificial intelligence detection of diabetic retinopathy	Retrospective cohort (n=893)	The AI system had a higher sensitivity for detecting mild to moderate DR than either general ophthalmologists or retina specialists compared with the clinical reference standard; It can potentially serve as a low-cost point-of-care diabetic retinopathy detection tool and help address the diabetic eye screening burden.
19	Ipp et al. 2021 [33]	Public health/ technology	Pivotal evaluation of an artificial intelligence system for autonomous detection of referrable and vision-threatening diabetic retinopathy	Prospective multicentre (n=942)	Improved access to accurate, reliable diabetic eye examinations may increase adherence to recommended annual screenings and allow for accelerated referral of patients identified as having vtDR.
20	Vujosevic et al. 2020 [34]	Public health	Strategies for DR screening	Systematic review (number of studies reviewed not disclosed)	Accessibility, affordability and quality of DR screening key to preventing vision loss; DR Screening using new technologies makes it more cost-effective
21	Pietris et al. 2022 [35]	Health economics	Health economic impact of implementing AI to ophthalmology in Australia	Systematic review (n=7)	AI is just as or more economically viable as human screening programmes
22	Tufail et al. 2017 [36]	Technology/health economics	Diagnostic accuracy and cost-effectiveness compared with human graders	Observational study (n=1,340)	Retmarker and EyeArt systems are cost-effective alternatives to manual grading alone; ARIAS have the potential to reduce costs in developed-world healthcare economies and to aid delivery of DR screening in developing or remote health care settings.
23	Ruamviboonsuk et al. 2021 [38]	Health economics	Economic evaluation of artificial intelligence in ophthalmology	Systematic review (n=7)	AI screening for DR, standalone or used with humans is more economically viable; Economic evaluation of AI for DR screening can be used as a model for AI to other ophthalmic diseases.
24	Barcelo et al. 2017 [39]	Public health/health economics	The cost of diabetes in Latin America and the Caribbean in 2015: evidence for decision and policy makers	Prevalence-based approach	Diabetes represented a major economic burden to the countries of Latin America and the Caribbean (LAC) in 2015; Trinidad and Tobago expends one of the highest amounts, per capita, in LAC on healthcare
25	Xie et al. 2020 [40]	Health economics	Artificial intelligence for teleophthalmology-based diabetic retinopathy screening in a national programme: an economic analysis modelling study	Prospective modelling (n=39,006)	There is a strong economic rationale for using deep learning systems as an assistive tool to screen for diabetic retinopathy; Estimated annual cost savings to the Singapore economy by 2050 would be $15 million annually

Leadership and change management 

Peifer et al. highlighted the need for strategic and transformational leadership when implementing AI systems, which feeds into the idea that leaders need to effectively communicate the vision and strategy of the proposed change to all stakeholders, including employees [41].

Stakeholders, including clinicians, managers, employees, and members of the public, will have to be convinced that the change to screening using AI would be in their best interests, with effective communication and support given during each step of the change management process. This would be especially sensitive for those who may be made redundant or expected to re-skill as a result of job loss via the newly automated process [17,18,32,35,38].

**Figure 2:**
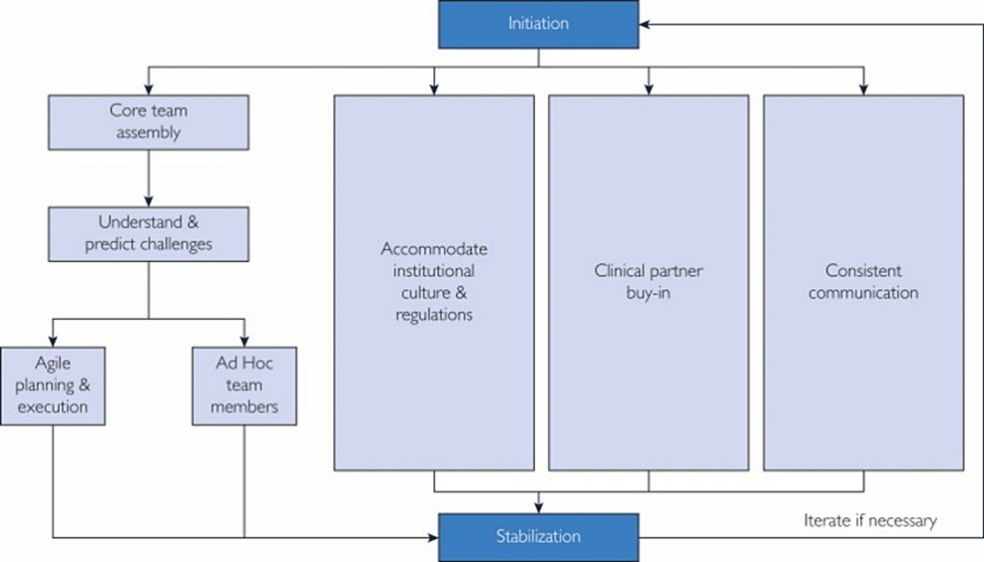
The Healthcare Novel Technology Change Management (HNT CM) model

Smith et al. excellently synthesized the principles of change management, including Kotter’s 8-step model of change and Lewin’s change model to develop the Healthcare Novel Technology Change Management (HNT CM) model [42]. This model addresses the unique needs and characteristics of novel technology implementation in healthcare systems and looks particularly at change management for AI. It can be modified and adapted as required (Figure 2).

## Conclusions

Many different AI systems have been approved for use for DR screening by the United States Food and Drug Administration, indicating that they have passed robust criteria for use. This systematic review identified multiple settings in which AI has either been used or modeled, using the widely accepted Markov model. These settings included low-, middle-, and high-income countries, as well as rural and urban settings within those countries. It found that AI for the purpose of DR screening was clinically as effective as, or superior to, human graders, confirming reliability, repeatability, and patient safety. Using AI for the purpose of DR screening confers tangible economic benefits in the form of cost savings via automation, as well as by modifying the natural history of the disease from early detection. This has a knock-on effect in preventing the onset and progression of other complications of diabetes and improving compliance with screening schedules. The economic benefits are most noticeable when AI or deep learning systems are deployed in low-income, rural settings.

Several gaps in the literature were identified, most notably surrounding the paucity of Trinidad and Tobago country-specific data on DR screening and the use of AI systems. Updated epidemiological and prevalence studies for DR and its impact are urgently required, while audit and prospective research following the implementation of an AI-based system for screening would help quantify the financial impact of the new screening system on the resources allocated to Ophthalmology in the public healthcare system in Trinidad and Tobago. The global prevalence of DR is expected to increase significantly in the coming decades with Trinidad and Tobago estimated to be disproportionately more affected by the burden of disease. The HNT CM model can be utilized specifically for change management for AI adoption in healthcare systems. It is estimated that, based on extrapolations from other settings such as Singapore, China, the United States, Thailand, and Rwanda, Trinidad and Tobago, as a high-income economy, stands to save approximately US$60 million annually (0.24% of GDP) from the implementation of an AI-based system to screen for DR.
